# Cell type-specific expression and subcellular localization of the human insulin upstream open reading frame (INSU) protein in pancreatic β-cells

**DOI:** 10.1016/j.jbc.2026.113209

**Published:** 2026-05-29

**Authors:** Qing-Rong Liu, Min Zhu, Lisa M. Hartnell, Jane Tian, Qin Yao, Xiaoming Zhong, Chee W. Chia, Paritosh Ghosh, Máire E. Doyle, Jennifer F. O’Connell, Josephine M. Egan

**Affiliations:** 1 Laboratory of Clinical Investigation, NIA/IRP/NIH, Baltimore, Maryland, USA; 2 Center of Excellence for Leukemia Studies, St Jude Children's Research Hospital, Memphis, Tennessee, USA

**Keywords:** insulin, islets, proteomics, diabetes, crinophagy, evolution

## Abstract

INSU was evolutionarily selected in human and chimp genomes by deletion of the 16 bp in *INS* 5′UTR regions present in other primate species that do not contain the upstream open reading frame. We now aim to identify the islet cell type in which INSU is produced, its subcellular distribution, and its response to stress in human islets. To do this, we developed an INSU specific rabbit polyclonal antibody, and we employed immunohistochemistry, immunofluorescence, and immuno-gold labeling with EM technologies. We used LC-MS/MS-based selected reaction monitoring proteomic assay to quantify INSU in islets, plasma and cerebral spinal fluid. Unlike mature insulin, INSU levels were unchanged in plasma and cerebral spinal fluid 2 hours after continuous intravenous glucose infusion. The INSU immunohistochemistry signal partially overlapped with that of insulin and was more intensely polarized than insulin in β-cells in islets. INSU was not present in α-, δ-, ε-, or PP-cells. Dual immunofluorescence and immuno-gold EM showed that INSU was present in immature insulin granules and crinosomes, but there was little to none present in mature secretory granules, implying INSU involvement in quality control of β-cells.

*INS* gene underwent selective, situational pressure during evolution as its expression migrated from neurons of invertebrates to the pancreas of vertebrates. Consequently, its functional spectrum diversified from its roles in development, learning-memory, and reproduction to metabolism in the Animalia kingdom (1). Human-specific genes (2, 3), promoters (4), exons (5), alternative splicing (6), lncRNAs (7) and miRNAs (8) are developmentally regulated and disproportionally more active in the human fetal neocortex (9); they are mostly inactivated in adulthood but can reemerge in aging tissues (10, 11, 12). The divergence between human and mouse lineages took place approximately 96 million years ago (MYA) (13) and the evolutionary adaptation resulted in genomic and epigenomic differences between insulin gene numbers, alternative splicing, and cell type-specific expression between the two species (14). Insulin has roles in embryonic cell survival and apoptosis during gastrulation and neurulation, dysregulation of its expression leads to embryopathies, and unprocessed proinsulin is required for embryonal survival (15). Human and Aves species diverged about 600 MYA (16), yet chicken has a single insulin gene and its embryonic-specific insulin upstream open reading frame (uORF) controls low-level and glucose-independent expression of proinsulin mainly in the neuroepithelial cells of the ectoderm, but also in mesoderm and endoderm layers (17).

At least 50% of human genes contain an alternative uORF which constitutes an added layer of gene regulation, functional diversity, and cellular stress responses (18, 19). Evolutionarily, human-specific uORFs are of recent origin and some of them became fixed by positive Darwinian selection (20). Any dysregulation of an uORF could potentially impact the expression and translation of the primary open reading frame (pORF) (21). Noncanonical uORFs are associated with human diseases, such as cancer, especially at the time of disease onset (22, 23). We recently uncovered multiple *INS* uORF isoforms encoding several microproteins that are driven from a promoter upstream of the canonical *INS* gene promoter (12). The uORF isoform transcripts begin from a transcription start site that is 273 bp upstream of the canonical *INS* mRNA cap-site and 90 bp downstream of the variable number tandem repeat region (IDDM2: insulin-dependent diabetes mellitus, a genetic locus two associated with type 1 diabetes mellitus (T1DM) susceptibility). In contrast, the canonical *INS* transcription start site is 362 bp downstream of the variable number tandom repeat site (24) resulting in the 5′-UTR of INSU isoforms overlapping with the conventional *INS* promoter. There are two in-frame N-terminal extended *INS* uORF isoforms generated by intron retention after the exon 1UC: the extended exon 1 of INSU1 retains intron one that is spliced to exons 2 and 3, the exon one of INSU2 retains intron 1 that spliced to exon 2 that retains intron 2 thereby creating an immature stop codon (14). Furthermore, there are three additional *INS* uORFs with their alternative upstream exon U1 directly spliced to the exon 2: INSUA uses the alternative exon 1UA and is translated to a truncated 53-AA peptide; INSUB uses the alternative exon 1UB and is translated in-frame with the pORF of preproinsulin to 153-AA peptide; INSUC uses the alternative exon 1UC and is translated to a truncated 73-AA peptide as shown in Figures 1 and 2 of our previous publication (14). What roles, if any, the protein products of the INSU isoforms play in nondiabetic and diabetic conditions have not yet been studied.

**Figure 1:**
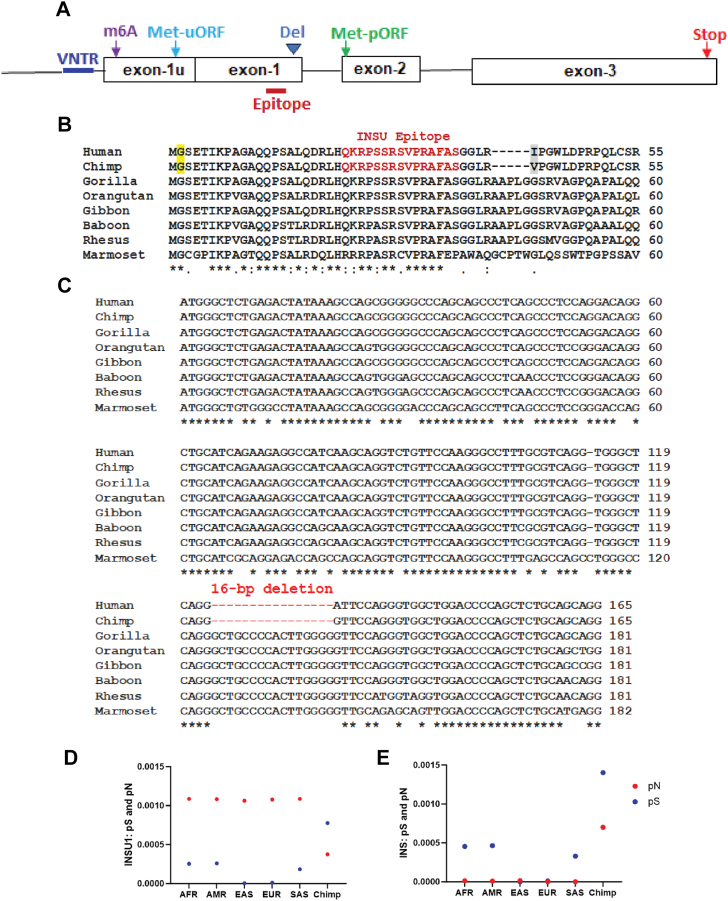
**Primate *INS* uORF gene structure and sequences.***A*, human *INS* gene structure: extended INSU exon-1u is shown in open box upstream of the canonical exon-1, variable number tandem repeats (VNTR) in *blue bar*, INSU mRNA m6A modification in *purple*, INSU translational initiation site Met-uORF in *cyan*, INSU human-chimp specific 16 bp deletion (Del) in *blue*, *INS* translational initiation site Met-pORF in *green*, stop codon in *re*d, and INSU epitope in *red horizontal bar*. *B*, amino acid sequence alignment of the uORF in primate species: potential myristoylation site is highlighted with *yellow*, the epitope peptide by *maroon lettering*, nonsynonymous amino acids between human and chimp highlighted with *gray*, gap between human-chimp from other primate by *dashed lines*, identical AA by *asterisks*, conservative AA by *semicolons*, and semiconservative AA by *colons*. *C*, the uORF coding nucleotide sequence alignment of primate INSU homologs: identical nucleotides are marked with *asterisks*. The 16 bp deletion in INSU of human and chimp is marked by *red dashed lines* and the label. *D*, scatter plot of pN and pS distribution of INSU1. *E*, scatter plot of pN and pS distribution of *INS* gene in different human populations and chimpanzees. AFR represents African; AMR Admixture American; EAS East Asian; EUR European; SAS South Asian. INSU, insulin upstream open reading frame; pORF, primary open reading frame; uORF, upstream open reading frame.

**Figure 2:**
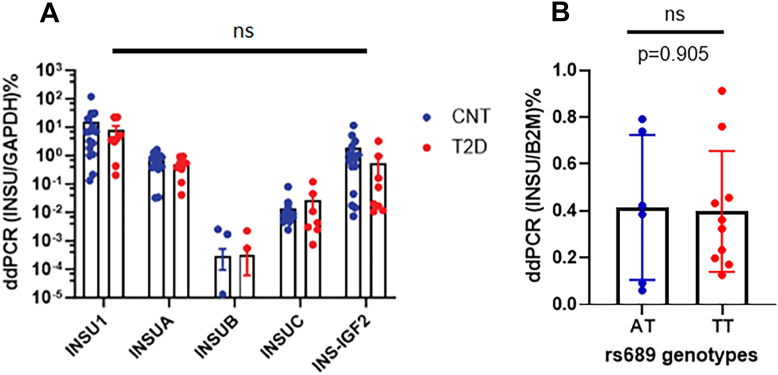
**INSU isoform mRNA levels in human islets (SEM) and their association with INS-VNTR genotypes.***A*, control (CNT, *n* = 15) and T2DM (*n* = 9) islet samples, at the mRNA levels, for INSU isoforms and INS-IGF2 fusion transcripts are similar. *B*, there is no association of INSU expression with INS-VNTR genotypes between rs689 (heterozygosity=0.39, global population) heterozygous (AT or class I/III) and homozygous (TT or class III/III) alleles. Homozygous (AA rs689) was not present in the genomic DNA of islet samples (*n* = 16). *Blue dots* are nondiabetic and *red dots* are T2DM islets. INSU, insulin upstream open reading frame; INS-VNTR, insulin variable number tandem repeat; T2DM, type 2 diabetes mellitus.

We revisited our previous findings related to INSU isoforms (14) by carrying out molecular, proteomic, β-cell stress response, anatomical, subcellular distribution analyses, immuno-electron microscopy, and by measuring INSU in human biofluids. We found that INSU is localized in immature secretory granules and crinosomes, associates with down-regulation of mature insulin translation in stress conditions, and its presence in plasma, unlike insulin, is not altered by glucose infusion.

## Results

### Evolutionary selection of human-chimp specific INS upstream ORF: INSU

We uncovered insulin uORF by searching 3544 human insulin expressed sequence tags (ESTs from pancreas, islets, and insulinomas), of which 20 ESTs aligned with the INSU exon 1u (Fig. 1*A*) connecting with intron 1 (14). The translational initiation site of INSU contains a consensus Kozak ribosomal binding site (25) (TGGGAGATGGGC) that is 45 bp upstream of the canonical *INS* mRNA 5′-cap-site (24). The Kozak score for INSU has a score of 0.87 in comparison to the canonical *INS* Kozak score of 0.93 (26). Therefore, we further extended the exon 1u to 5′ of the canonical *INS* exon one and added an additional translational initiation codon of Met-uORF upstream of the canonical Met-pORF (Fig. 1*A*). We used SRAMP algorithm to predict the potential N^6^-methyladenosine (m^6^A) RNA modification and found that the 5′UTR of INSU nucleotide sequence contains very high confidence (GGACA, combined score = 0.668) of m^6^A mRNA methylation sites (27) (Fig. 1*A*) and SignalP 6.0 (28) predicted no signal peptide of INSU isoforms. Using algorithm of N-terminal N-Myristoylation of Proteins (29), we predict that the second glycine after Met-uORF contains an N-myristoylation site (score −1.462), potentially allowing its membrane attachment (Fig. 1*B*, the second G with yellow highlight). We aligned INSU peptide and nucleotide sequences of human and chimp (*Pan troglodytes*, NC_03689 0.1, diverged 6 MYA) (30) with other primate species and found a 16 bp deletion in the human-chimp ORF region, generating an INSU protein coding region that is disrupted by a frame shift in other primate species (Fig. 1, *B* and *C*). The INSU homologous sequences (Fig. S1: phylogenetic tree) of gorilla, orangutan, gibbon, rhesus monkey, baboon, and marmoset (13) do not contain the 16 bp deletion (Fig. 1*C*), therefore, lost the peptide coding potentials by frameshift. We found no significant conservation of the uORF sequences outside of primate order and the corresponding mouse *Ins1* and *Ins2* upstream regions do not contain Kozak sequence and uORF (Fig. S5).

We performed evolutionary analysis using SNPgenie program (31) to determine the evolutionary trajectory of the INSU1 and *INS* (14, 31). We calculated the synonymous substitution rate (pS) and the nonsynonymous substitution rates (pN) of 661 Africans (ARF), 347 Admixed Americans (AMR), 504 East Asians (EAS), 503 Europeans (EUR), and 489 South Asians (SAS) (32) compared to those of 25 chimps (Table S1) (33). A pN/pS ratio >1 predicts positive selection favoring nonsynonymous SNPs and <1 predicts purifying selection removing nonsynonymous SNPs faster than synonymous polymorphisms (31). We found that INSU1 is likely to be under positive selection in human populations (pN > pS) (Fig. 1*D*), while chimp is under purifying selection (pS > pN) like that of *INS* (Fig. 1*E* and Table S1). The uORFs of INSU1, INSUA, INSUB, and INSUC isoforms coding sequences were predicted to be positively selected (Fig. S2, *A*–*D*) for ARF, AMR, EAS, EUR, and SAS populations.

### INSU isoform mRNA levels in nondiabetic and diabetic pancreatic islets

We designed TaqMan primers and probes for sequences that overlap the upstream translation initiation codon of INSU (see Methods) and the spliced junctions (exon 1UA, 1UB, and 1UC, and intron one retention site) and INS-IGF2 fusion transcript (14) to measure mRNA levels in isolated human islets. A two-tailed unpaired *t* test of RT-qPCR data revealed that there were no significant changes at mRNA levels for INSU1 (*t*_(21)_ = 0.79, *p* = 0.37), INSUA (*t*_(21)_ = 1.21, *p* = 0.29), INSUB (*t*_(21)_ = 0.02, *p* = 0.94), INSUC (*t*_(18)_ = 0.90, *p* = 0.38), and INS-IGF2 (*t*_(21)_ = 0.14, *p* = 0.27) in type 2 diabetes mellitus (T2DM) islets (*n* = 9) compared to nondiabetic islets (CNT, *n* = 15) (Fig. 2*A*). *INS* class I shorter allele (26–63 repeats) provides susceptibility while the class III longer allele (141–209 repeats) is protective of T1DM (34). We did not find any change in INSUA expression (Fig. 2*B*) with class I and class III alleles of insulin gene variable number tandem repeats (INS-VNTR) in islet samples (*n* = 16) analyzed with Chi-square test (X2 [1, *n* = 16] = 1.63, *p* = 0.202); therefore we can conclude that INSU expression is not associated with insulin-dependent diabetes mellitus 2 (IDDM2) INS-VNTR genotypes.

### INSU levels in plasma and cerebral spinal fluid (CSF) are independent of circulating glucose levels

**Figure 3:**
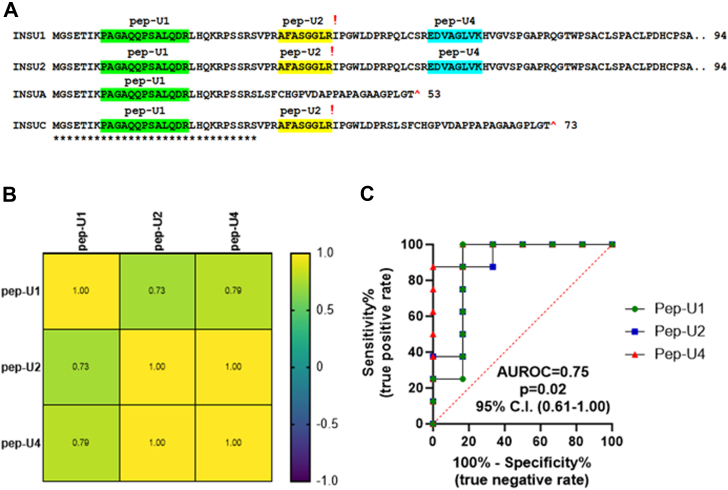
**INSU peptide isoforms and correlation.***A*, pep-U1 (*green*), pep-U2 (*yellow*), and pep-U4 (*cyan*) are highlighted. The amino acids numbers are indicated at the right. *Red exclamations* above the amino acid sequences indicate the 16 bp deletion sites that created uORF in human and chimp INSU isoforms. *B*, correlation matrix of pep-U1, -U2, and -U4 in human islets. *Yellow gradient* represents degree of correlation. *C*, the diagnostic accuracies analysis of pep-U1, -U2, and -U4 were calculated by area under the receiver operating characteristic curve. The representative pep-U4 AUROC was shown in Figure 3*C*. INSU, insulin upstream open reading frame; uORF, upstream open reading frame.

**Figure 4:**
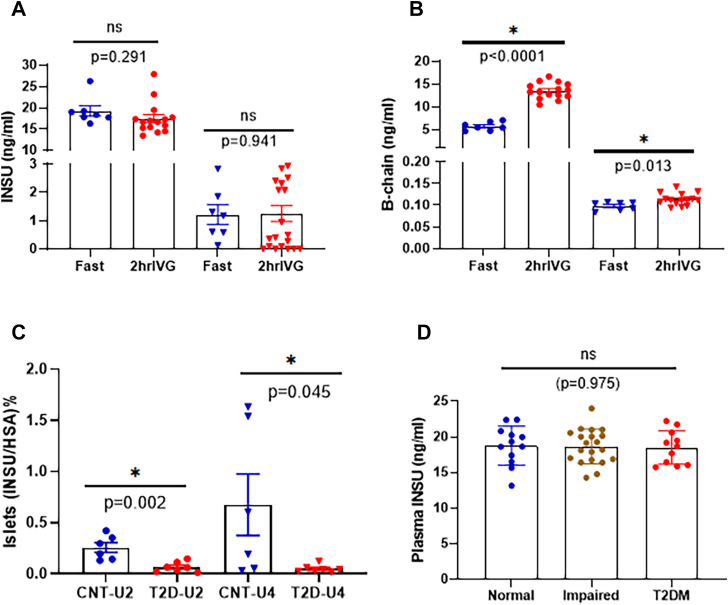
**SRM quantification of INSU in islets, CSF, and plasma.***A*, INSU peptide levels were comparable in both fasting (*n* = 7, *blue*) and after 2 h continuous intravenous glucose clamp (*n* = 15, *red*) in plasma (*circles*) and CSF (*triangles*) samples. *B*, insulin B-chain (pep-B1) was highly responsive to 2 h of continuous intravenous glucose clamp. *C*, the INSU pep-U2 (*circles*) and -U4 (*triangles*) were significantly reduced in T2DM (*red*) compared to nondiabetic (*blue*) islets. *D*, INSU peptide levels were comparable in fasting plasma samples from normal (*n* = 12, *blue*), impaired (*n* = 20, *brown*), and T2DM (*n* = 11, *red*) subjects. CSF, cerebral spinal fluid; INSU, insulin upstream open reading frame; SRM, selected reaction monitoring assay; T2DM, type 2 diabetes mellitus.

We developed MS based-SRM (14) for INSU-derived peptides of pep-U1, -U2, and -U4, targeting different INSU isoforms, pep-U1 aligns with all the INSU isoforms, pep-U2 with INSU1, INSU2, INSUC isoforms, and pep-U4 with INSU1 and INSU2 isoforms (Fig. 3*A*). We observed high correlation coefficients among INSU microproteins in islets, that is, between pep-U1:U2 (*r* = 0.73, *p* = 0.003), pep-U1:U4 (*r* = 0.79, *p* = 0.001), and pep-U2:U4 (*r* = 0.99, *p* < 0.0001) (Fig. 3*B*). The SRM diagnostic accuracy in normal and T2DM islets was also highly correlated, pep-U1 (AUROC=0.875, *p* = 0.020), -U2 (AUROC=0.875, *p* = 0.020), and -U4 (AUROC=0.875, *p* = 0.020), and US (AUROC=0.875, *p* = 0.020) (Fig. 3*C*). We selected pep-U4 that has the best transition and high expression as a representative SRM peptide to determine INSU levels in islets, plasma and cerebral spinal fluid (CSF) samples. INSU peptide level was 14.4 ± 2.7 fold higher in plasma compared to CSF samples. Using two-tailed unpaired *t* test, we did not find a significant change (Fig. 4*A*) of INSU levels in plasma (*t*_(20)_ = 1.08; *p* = 0.29) and CSF (*t*_(26)_ = 0.07; *p* = 0.94) between fasting and after 2 h of continuous intravenous glucose infusion while the pep-B1 derived from B-chain was highly glucose responsive (Fig. 4*B*) in plasma (*t*_(20)_ = 11.07; *p* < 0.0001) and CSF (*t*_(20)_ = 2.72; *p* = 0.013) (14). This shows that INSU secretion is not altered by glucose stimulation nor cleared as efficiently as is insulin from plasma. We measured the INSU SRM pep-U2 and -U4 peptide levels in islets from T2DM (*n* = 9) and nondiabetic (*n* = 15) postmortem pancreata, and we found that INSU peptides were significantly reduced in T2DM islets, compared to nondiabetic islets. Based on two-tailed unpaired *t*-tests there was significant reduction in pep-U2 (*t*_(11)_ = 3.93, *p* = 0.002) and -U4 (*t*_(11)_ = 2.27, *p* = 0.045) (Fig. 4*C*) in T2DM islets. However, INSU levels in fasting (>12 h) plasma from nondiabetic (*n* = 12), impaired (*n* = 20) and T2DM (*n* = 11) in BLSA subjects (Fig. 4*D* and Table S2) were not statistically different by one way ANOVA analysis (F_(2,40)_, *p* = 0.98). glycated hemoglobin (*r* = −0.111, *p* = 0.488), body mass index (*r* = 0.025, *p* = 0.876), and age (*r* = 0.087, *p* = 0.577) were not correlated with INSU peptide levels by Spearman correlation analysis.

### INSU subcellular localization in human β-cells in islets

**Figure 5:**
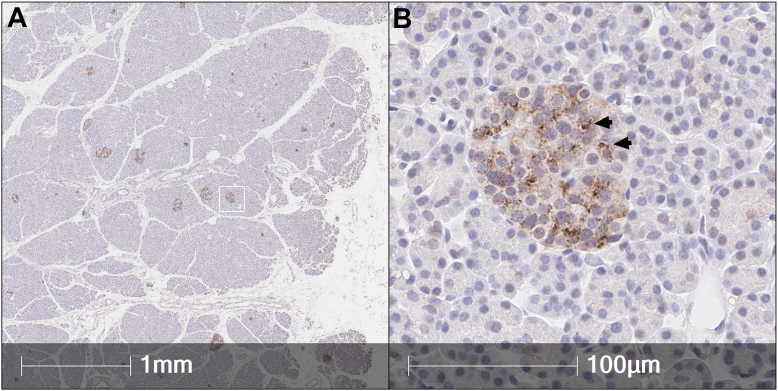
**Immunohistochemistry localization of INSU in whole human pancreas.***A*, DAB (3,3′-Diaminobenzidine) IHC (1 mm) staining with INSU rabbit polyclonal antibody in human pancreas and *B*, the enlarged panel (100 μm); *black arrows* indicate intensely polarized INSU staining in β-cells. IHC, immunohistochemistry; INSU, insulin upstream open reading frame.

**Figure 6:**
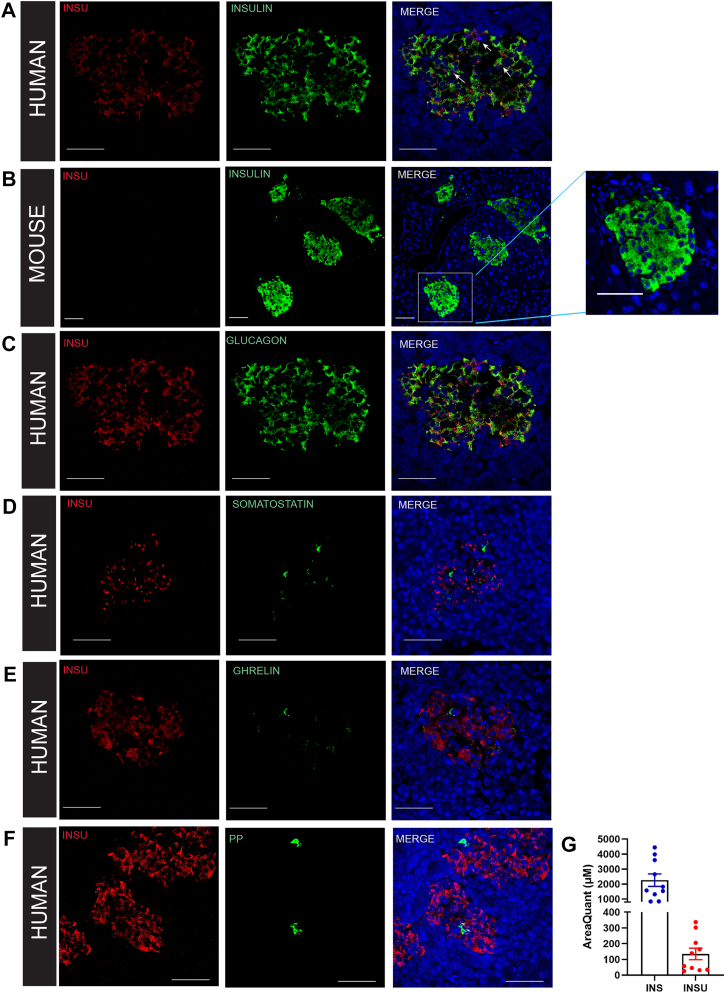
**Immunofluorescence localization of INSU with islet cell type markers in whole pancreas.***A*, dual immunofluorescence (IF) staining of insulin (*green*) and INSU (*red*) in a human pancreas. *White arrows* indicate intensely polarized INSU staining in b-cells. *B*, dual IF staining of insulin (*green*) and absence of INSU (*red*) in a mouse pancreas. *C*, dual IF staining of nonoverlapping glucagon (*green*) and INSU (*red*), *D*, nonoverlapping somatostatin (*green*) and INSU (*red*), *E*, nonoverlapping ghrelin (*green*) and INSU (*red*), *F*, nonoverlapping PPY (*green*) and INSU (*red*) in human islets. *White scale bars* represent 10 μm. *G*, cytoplasmic levels of INSU, proinsulin, and insulin in β-cells (quantified in 10 human islets) using HALO Area Quant Fluorescence module. INSU, insulin upstream open reading frame.

We developed (12) and validated an INSU rabbit polyclonal antibody by islet specific immunofluorescent staining in human pancreas that was blocked by incubating with 5 times excess of the antigenic peptide (Fig. S3*A*) and not by equal molar concentration of mature insulin (Fig. S3*B*) (Humulin R, Eli Lilly), both at RT for 30 min. Using immunohistochemistry (IHC), we found that INSU rabbit polyclonal antibody stained only islets and no other part of human pancreas (Fig. 5*A*). We also found that INSU IHC staining was intensely polarized (Fig. 5*B*, black arrows). Using dual immunofluorescence (IF), it appeared that INSU rabbit polyclonal antibody localized within insulin containing cells. Interestingly, the intensely polarized INSU did not colocalize with insulin (Fig. 6*A*, white arrows) and as expected, INSU was not present in mouse islets (Fig. 6*B*). We did not find INSU in glucagon (Fig. 6*C*), somatostatin (Fig. 6*D*), ghrelin (Fig. 6*E*) or pancreatic polypeptide (Fig. 6*F*) positive cells. Using HALO area quantification module, INSU positivity amounted to 5.2 ± 0.8% (*n* = 10 islets) of the total proinsulin and insulin present in β-cells (Fig. 6*G*). Furthermore, INSU was present in 25.2 ± 4.3% of cells that also contained insulin (Fig. S4): approximately 60% of total cells in adult human islets are β-cells (35). Therefore, INSU is β-cell specific, has a different subcellular localization from insulin and was present in a quarter of all β-cells.

**Figure 7:**
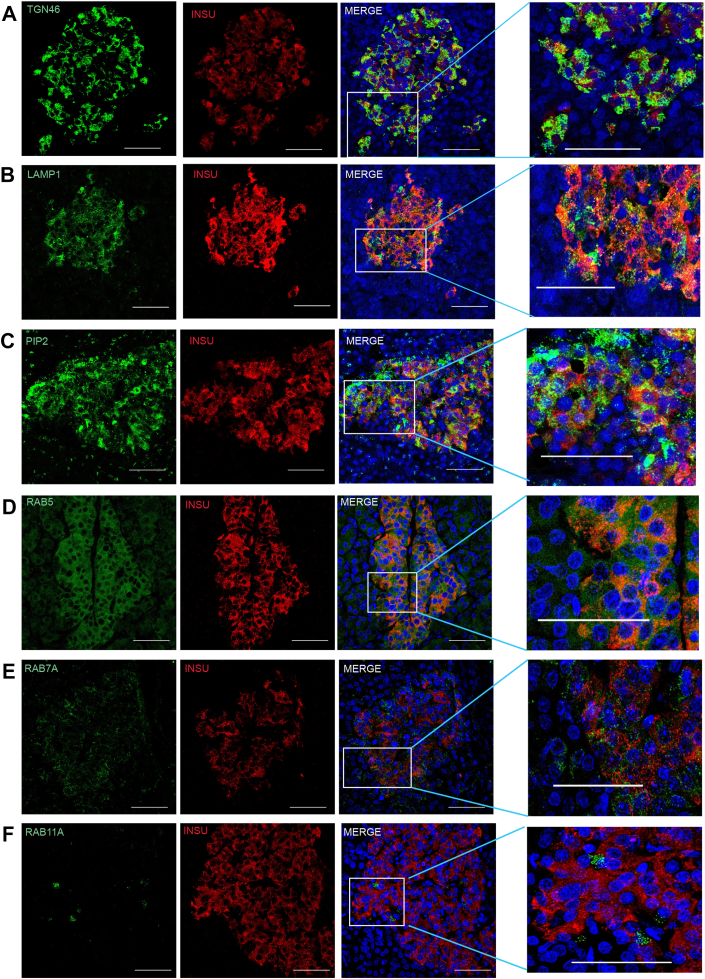
**Immunohistochemistry localization of INSU using organelle markers in human pancreas.***A*, Dual IF staining of INSU with overlapping TGN46 (trans-Golgi network), *B*, LAMP1 (lysosome), *C*, PIP2 (plasma membrane), *D*, RAB5A (early endosome), *E*, RAB7A (late endosome), *F*, nonoverlapping RAB11A (recycling endosome). The *right panels* are the enlarged images of the *white frames* to the *left*. *White scale bars* represent 20 μm. INSU, insulin upstream open reading frame.

We further investigated the INSU intracellular organelle association and found INSU to be present in TGN46 labeled trans-Golgi network (Fig. 7*A*), in LAMP1 labeled lysosomes (Fig. 7*B*), in PIP2 labeled membrane bilayers (Fig. 7*C*), in RAB5A labeled early endosomes (Fig. 7*D*), in RAB7A labeled late endosomes (Fig. 7*E*), but not in RAB11A labeled recycling endosomes (Fig. 7*F*), indicating that INSU might be involved in the endosome and lysosomal trafficking, and signaling pathways.

**Figure 8:**
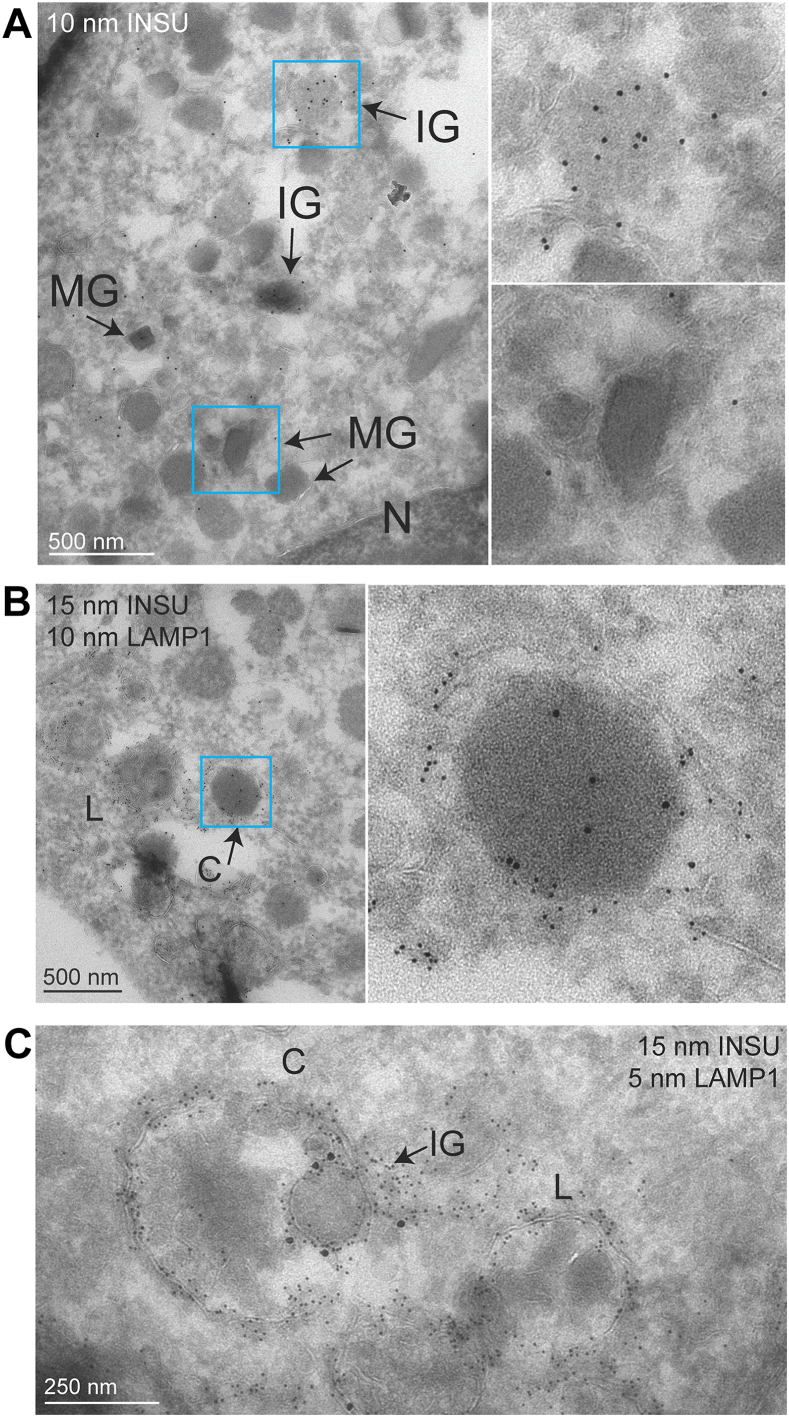
**Electron micrographs show INSU localization in some of the immature granules and lysosomes.***A*, INSU localization in immature granules (IG) labeled by 10 nm protein A-gold (*arrow*) but not in mature granules (MG); enlarged insets in *blue border* on the *upper* and *lower right*, respectively. Both immature granules (IG) with *arrows* are positive for INSU with the darker of the two under a slight fold in the section. Note that the immature granules are dense and more crystalline, whereas the immature granules are amorphous and less dense. *B*, 15 nm INSU and 10 nm LAMP1 colocalization in a crinosome, *blue box* enlarged on *right*. *C*, INSU represented by 15 nm *gold* and LAMP1 represented by 5 nm *gold*. Note early phase of crinosome (C with *arrow*) positive for LAMP1engulfing an INSU-labeled IG (*arrow*) with other lysosomes nearby (L). Crinosome (C), nucleus (N), lysosome (L). INSU, insulin upstream open reading frame.

Using immunoelectron microscopy performed on thawed, PFA fixed, ultrathin cryo-sections of isolated human islets, INSU, visualized with either 10 or 15 nm protein A-gold, was localized in immature secretory granules (>5 gold particles per granule) but little was present in mature secretory granules (Fig. 8*A*). Moreover, INSU and LAMP1 antibodies labeled crinosome-like structures in INSU positive immature granules surrounded by a lysosomal membrane (Fig. 8*B*). Furthermore, INSU and LAMP1 antibodies labeled an early phase of INSU positive immature granules engulfed by a lysosome (Fig. 8*C*) in the process of crinophagy.

### Differential responses of INSU and insulin to β-cell stress

**Figure 9:**
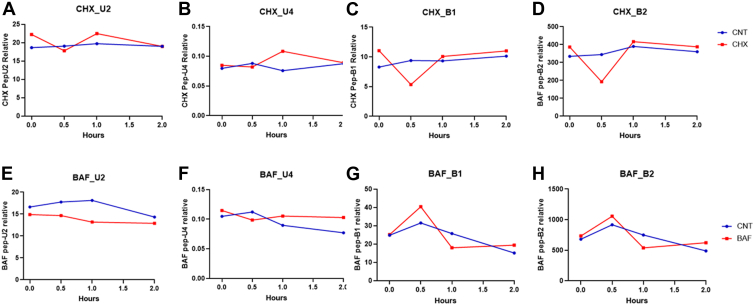
**Human islets, treated with cycloheximide and bafilomycin.** Cycloheximide (10 μM) for 0.5, 1, and 2 h, followed by SRM assay of *A*, pep-U2, *B*, pep-U4, *C*, pep-B1, and *D*, pep-B2; and bafilomycin (0.1 μM) for 0.5, 1, and 2 h, followed by SRM assay of *E*, pep-U2, *F*, pep-U4, *G*, pep-B1, and *H*, pep-B2. *Red line* represents the drug treatment and *blue line* the vehicle treatment. SRM, selected reaction monitoring assay.

Cycloheximide is reported to cause cell stress at early points after cellular treatment (36) and we found that in the presence of cycloheximide (10 μM) the pep-U2 and -U4 levels increased at 1 h in contrast to the pep-B1 and -B2 (correlation coefficient: *r* = 0.98, *p* < 0.0001) that had decreased by 0.5 h and recovered 1 h (Fig. 9, *A*–*D*), implying that INSU is stress-induced in β-cells. Bafilomycin A1 (0.1 μM) is reported to inhibit autophagy in the late phase (37), and we found that the pep-U2 and -U4 levels were decreased up to 2 h in contrast to the pep-B1 and -B2 levels that had increased by 0.5 h and then had subsequently declined by 1 and 2 h (Fig. 9, *E*–*H*), implying inhibition of lysosome function in β-cells had more effect on mature insulin than INSU.

## Discussion

We uncovered human-chimp specific insulin uORFs and validated the expression of INSU isoforms in human islets at the mRNA level by the EST alignment and RT-qPCR using TaqMan probes that hybridized specifically to the INSU translational initiation methionine codon. We verified INSU at the peptide level in biofluids by quantitative proteomics; and at the anatomic level in human pancreas by IHC and Immuno-EM using a custom-made INSU specific rabbit polyclonal antibody. The uORF sequence is not present in the Cross-Tissue Cartography GTExPortal (https://gtexportal.org/home/) database because the RNAseq analysis of human tissues is not yet of sufficient depth to detect these rare transcripts within β-cells that are a miniscule subpopulation of the total human pancreas.

Compared to insulin, INSU secretion was not influenced by circulating glucose levels and duplex IF quantification showed that INSU was 5.2 ± 0.8% of total proinsulin- and insulin-containing mass in islets. Although INSU intracellular concentration is low relative to insulin, its effects may be felt throughout the β-cell. However, in plasma, INSU levels are relatively high compared to that of insulin (14, 38), which is rapidly internalized and degraded (half-life about 4–11 min) through insulin receptor binding, internalization, and finally degradation (39). The INSU peptide may not bind to insulin receptor and, similar to C-peptide, is likely eliminated through renal clearance. Furthermore, INSU microproteins in human plasma might potentially be autoantigenic and recognized as self-peptides. For instance, defective insulin ribosomal products INS-DRiP (40) and INS-Splice (41) isoforms are involved in T1DM autoantigen activation of cytotoxic T cells. Using NetMHC 4.0 algorithm (42), we found that the INSUA 9mer peptides APAGAAGPL and PSSRSLSFC have high affinity binding at concentrations of 4.15 and 5.66 nM, respectively, to HLA-B0702, which are in the range of the known autoantigenic insulin signal peptide epitope HLVEALYLV (18.31 nM) and B-chain epitope LYLVCGERG binding to HLA-A0201 (6.95 nM) (43). Therefore, INSU isoforms potentially are autoantigens.

Currently, the regulatory mechanisms and functions of human INSU microproteins are unknown. One possibility is that INSU regulates insulin translation. Insertion of uORF into 5′UTR of the rat insulin gene construct slowed the translation rate of preproinsulin (44). Translation of INSU might be independent of the 7-methylguanosine (m^7^G) cap translation of canonical insulin (24) since the mRNA m^6^A modification of the INSU 5′UTR could initiate m^6^A-mediated translation (45). The INSU mRNA m^6^A modification represents one of ∼7000 mRNA species that are reversibly modified by m^6^A methyltransferase-3 (METTL3), mediating RNA stability, translation, splicing, and export (46) – all of which play important roles in β-cell development (47).

We attempted to measure the half-life of INSU in human β-cells by performing SRM on INSU levels in *postmortem* human islets treated with cycloheximide and bafilomycin. However, our attempts were confounded by the necessity to do the SRM in serum free medium (a requirement of SRM) leading to nutrient deprivation of the islets, a nonideal condition and the large heterogeneity between human islet batches. Human insulin inside β-cells has a low turnover rate and long half-life lasting days (48) while uORF turnover rates are generally shorter lasting a few hours inside cells (49). In a short period of time; however, we found that upregulation of INSU attenuated insulin translation in the initial phase of cycloheximide stress (50). Furthermore, we found that inhibition of lysosomes led to increased insulin level at 0.5 h while INSU level steadily declined during bafilomycin treatment. The results point to INSU leading to down-regulation of the translation of insulin (51) as part of an integrated stress response (52) that involves eIF2α-pS51 phosphorylation by stress-induced kinases, such as PKR, PKR-like endoplasmic reticulum kinase, general control nondepressible two and double stranded RNA dependent protein kinase (GCN2), and heme-regulated eIF2α kinase (53), thereby reducing insulin translation during β-cell stress. We propose that INSU was beneficially selected to cope with β-cell stress induced by dietary changes during human evolution and the reduced INSU microprotein levels in T2DM islets increases the β-cell vulnerability toward stress.

This SRM assay utilized human islets from two donors for each drug treatment, which constrains the number of biological replicates and the statistical power available to draw definitive conclusions. This reflects the inherent challenges of working with primary human islet tissue, including restricted availability and significant interdonor variability in islet composition and function. Accordingly, the findings presented here should be considered hypothesis-generating rather than confirmation. However, the analytical approach employed partially compensates for this limitation. The multiplexed mass-based SRM method in biological duplex measures the highly correlated INSU and B-chain peptides simultaneously within the same run of the same sample, eliminating run-to-run variability and ensuring that all peptides experience identical sample preparation, ionization conditions, and instrument variation. Two INSU tryptic peptides, pep-U2, and -U4 representing alternative spliced INSU peptides, and two insulin tryptic peptides, pep-B1 and -B2 representing mature B-chain of insulin (14), likely reflect genuine biological covariation rather than a technical artifact.

Plasma levels of INSU peptide measured by SRM were not altered by 2 h of intravenous sustained hyperglycemia in contrast to the SRM insulin level that is highly responsive to glucose (14). Moreover, INSU levels were similar in the plasma of nondiabetic, glucose-impaired, and T2DM subjects. The INSU promoter lacks the conventional promoter region of the *INS* pORF that includes the proximal TATA, GG, A, C, E, CRE box *cis*-acting elements which bind to the transcription factors, PDX1, MAFA, NEUROD1, and CREB (54), and therefore glucose and incretin regulation of INSU is absent (15). In addition, INSU has no signal peptide, and its secretion is not through a conventional ER-to-Golgi-to-secretory vesicle route because by EM it is not present in mature insulin-containing secretory vesicles. Its secretion is likely though unconventional autophagosome-mediated protein secretion (55) and/or endosome and multivesicular body mediated exosome secretion (56). Indeed, we recently observed that INSU is located on the surface of exosomes isolated from cultured medium of human islets, as well as from human plasma (unpublished observations).

We propose that INSU is involved in the quality control of immature granules through endolysosome-mediated crinophagy (57) since it is present in early and late endosomes and lysosomes. Proinsulin-containing immature granules are amorphic and noncrystalline with less of a halo that is present in the classical EM appearance of insulin secretory vesicles (58), and therefore INSU likely ‘tags” impaired immature granules fusion to lysosomes for degradation and recycling (59).

Pancreatic β-cells are one of the most vulnerable cell types because the high and fluctuating demands for insulin transcription and translation hinders the synthesis of gene products that would protect it from reactive oxygen species and the ER unfolded response; β-cells, for example, contain very low levels of catalase that would be protective against reactive oxygen species (60). INSU may have evolved from “meat-adaptive” or “beneficial” uORFs selected for the dietary alterations of omnivorous humans and chimps because the other great apes and primates are largely herbivorous (61). For instance, carnivorous feline species lack the peptide hormone nesfatin-1 in their β-cells while it is highly expressed in β-cells of herbivores (62). INSU under the positive selection (pN > pS) in human populations and not in chimps might represent a rapid evolutionary change in human populations about 4 MYA as they adapted to a huge increase in metabolic demand from the transition from chimp quadrupedalism to human bipedalism (63, 64). Moreover, human-chimp INSU isoforms likely function as growth factors in early embryo development. Chicken embryo-specific insulin uORF regulates gastrulation and neurulation independent of glucose levels (17). It is possible that the interactions between INSU and insulin during neurulation and neuronal differentiation in the embryonal state (65) contribute to the human-specific phenotypes of a larger neocortex, more meat consumption (63), and longevity (9, 66). In fact, human aging is an adaptive phenotype under selection (67) and the associated genes often demonstrate relatively high rates of polymorphism (68). For instance, as regards *APOE*, chimps have just the *ε*4 allele, whereas humans have *ε*3 which arose approximately 220,000 and the *ε*2 allele which arose approximately 80,000 years ago (69). These new alleles proved evolutionarily robust in mitigating against the risk *ε*4 allele of Alzheimer’s disease (70).

In summary, the human INSU isoforms are regulated by transcription, translation, alternative splicing, RNA surveillance, protein modification and degradation, and intracellular protein sorting - all of which might play important roles in embryogenesis, metabolism, β-cells adaptation, and quality control under stress conditions.

## Experimental procedures

### Sources of clinical samples

We measured INSU peptide levels in plasma samples of nondiabetic adults after a 12-h overnight fast (fasting, *n* = 7) and 2 h after continuous intravenous glucose (2 hr-IVG, *n* = 15) administration whereby circulating glucose was continuously clamped at fasting levels plus 98 mg/dl (71). In addition, we obtained the matched CSF samples after the 2 hr-IVG. We obtained fasting plasma samples from the Baltimore Longitudinal Study of Aging (BLSA) (72, 73) from normal controls (*n* = 11), glucose impaired (*n* = 20) and T2DM patients (*n* = 12) for INSU quantification. National Institutes of Health Institutional Review Board abides by the Declaration of Helsinki principles. The clinical protocols have institutional review board approval (National Institutes of Health: 03-AG-0325 and 15-AG-0063). Fasting CSF samples (*n* = 7) were obtained from an IRB-approved study (10-AG-0423/CR002894), a separate study from the previous two listed (74). Human control (15 subjects) and T2DM (9 subjects) fresh islets were received from Integrated Islet Distribution Program (IIDP). We also obtained two control frozen islet samples from IIDP included in the genotyping experiment. Formalin-fixed paraffin-embedded (FFPE) human pancreas blocks were *postmortem* samples from a 24-year-old male (for DAB staining, Fig. 5, *A* and *B*) and a 46-year-old female (for IF staining, Figs. 6, *A*–*F* and 7, *A*–*F*) provided by United Network for Organ Sharing (UNOS, Richmond, VA).

### RNA isolation, cDNA synthesis, RT-qPCR, genotyping, and bioinformatics

Total RNAs were extracted from human islets using Trizol (Thermo Fisher Scientific) protocol. Single strand cDNA was synthesized from total RNA using qScript XLT cDNA SuperMix (Quantabio). For quantitative real-time PCR assessments, INSU1 specific TaqMan probes were designed specifically to the uORF and intron1 retention region, along with INSU2 to the intron two retention region, INSUA to exon 1UA to exon-2, INSUB to exon 1UB to exon2, INSUC to exon 1UC to exon 2, INS-IGF2 to the junction of exons of *INS* and *IGF2*, and *INS* to the common exon-2 (14). Custom TaqMan probes were from Thermo Fisher Scientific. Droplet Digital PCR (ddPCR) absolute values were derived from Poisson distribution of positive and negative droplets (QX200 ddPCR System, Bio-Rad) that were normalized with an endogenous control, β2 microglobulin (*B2M* Vic-labeled, Cat# 4326319E) or glyceraldehyde-3-phosphate dehydrogenase (*GAPDH* Vic-labeled, Cat# 4325792). Genomic DNA of islet tissues were isolated using QIAamp DNA Blood Mini Kit (Cat#: 51104). SNP rs689 A-allele and T-allele are in complete linkage disequilibrium with INS-VNTR classes I (ACAGGGGTGTGGGG repeated 28–44 times) and III (ACAGGGGTGTGGGG repeated 138–159 times), respectively (75). We carried out the allelic discrimination assay for the available genomic DNA samples (76) of islets (*n* = 16) that have the matching ddPCR values in our islet samples and the results were analyzed using TaqMan Genotyper software (Thermo Fisher Scientific). EMBL-EBI Clustal Omega algorithm was used for sequence analysis (77). Homonid *INS* gene polymorphism data were downloaded from the 1000 Genomes Project (https://www.internationalgenome.org/) (32) and the Great Ape Genome Project (https://eichlerlab.gs.washington.edu/greatape/) (33). SNPGenie (31) was used to estimate the average number of pairwise SNP substitutions per site at nonsynonymous (pN) and synonymous (pS) coding sites in the INSU1 and preproinsulin and to predict the directions of natural selection on INSU1.

### IHC of INSU in pancreas

FFPE human and mouse pancreas blocks were sectioned (10 μm) using a microtome (RM2255, Leica Biosystems) onto ColorFrost Plus Microscope Slides (Cat# 1255016, Fisher Scientific). IHC of pancreas sections with the INSU rabbit polyclonal antibody (1:100 dilution) by DAB (3,3′-diaminobenzidine) staining was performed by Histoserv Inc. Dual IF was performed on human and mouse FFPE pancreatic sections using sodium citrate buffer antigen retrieval solution (pH 6.0; Cat# C9999, Sigma-Aldrich) at 96 °C for 30 min. See Table 1 for list of islet cell type antibodies. Table 1 also lists subcellular membrane structure markers for dual INSU IF assays, we used mouse monoclonal antibodies to TGN46 (trans-Golgi network), LAMP1 (lysosome), PIP2 (plasma membrane), RAB5A (early endosome), RAB7A (late endosome), and RAB11A (recycling endosome) (78). Confocal fluorescence images were captured using a Zeiss LSM-880 confocal microscope (Carl Ziess). The quantification and localization of INSU and insulin in islets were analyzed using HALO Area Quantification FL module and Object Colocalization FL module (Indica Labs). All the images underwent the same adjustments prior to the final quantification by the modules.

**Table 1:** List of commercial antibodies used in this work

Antigen	Host species	Dilution	Manufacturer	Catalog and RRID	Location
INSU	Rabbit/pAB/IgG	1/100	GeneMed Synthesis Inc.	Custom-made	San Antonio, TX
Insulin	Mouse/mAB/IgG1	1/500	Sigma-Aldrich	I2018/AB_260137	Saint Louis, MO
Glucagon	Guinea pig/pAB/IgG	1/100	Linco Research Inc.	4031-01F/AB_433707	Saint Charles, MO
Ghrelin	Goat/pAB/IgG	1/100	Santa Cruz Biotechnology	sc-10368/AB_2232479	Santa Cruz, CA
STS	Mouse/mAB/IgG1	1/100	Santa Cruz Biotechnology	sc-74556/AB_2271061	Santa Cruz, CA
PPY	Mouse/mAB/IgG2b	1/100	Santa Cruz Biotechnology	sc-514155/N.A.	Santa Cruz, CA
LAMP1	Mouse/mAB/IgG1	1/100	Santa Cruz Biotechnology	sc-20011/AB_626853	Santa Cruz, CA
PIP2	Mouse/mAB/IgM	1/100	Santa Cruz Biotechnology	sc-53412/AB_630097	Santa Cruz, CA
RAB5A	Mouse/mAB/IgG2b	1/100	Santa Cruz Biotechnology	sc-46692/AB_628191	Santa Cruz, CA
RAB7A	Mouse/mAB/IgG1	1/100	Santa Cruz Biotechnology	sc-376362/AB_10987863	Santa Cruz, CA
TGN46	Mouse/mAB//IgG1	1/100	Thermo Fisher Scientific	MA3-036/AB_325484	Hunt Valley, MD
Rabbit anti-mouse	Rabbit/pAB/IgG	1/50	Jackson ImmunoResearch	315-001-003/AB_2340038	West Grove, PA
RAB11A	Mouse/mAB//IgG1	1/100	Thermo Fisher Scientific	MA5-37686/AB_2897610	Hunt Valley, MD

### Electron microscopy with INSU-immunogold labeling in islets

Immunogold EM was performed on isolated islets (SAMN35848421) from human pancreas. Isolated islets were fixed in 4% paraformaldehyde in 100 mM sodium phosphate buffer (pH 7.4) for 15 min then pelleted in the fixative for 2 min, 700 rpm in a microfuge. The pellets were rinsed in 0.1% fish skin gelatin in PBS and centrifuged as before, rinsed 3 times for 5 min per rinse, then resuspended in 5% gelatin, and set in ice. Once set, the gelatinized islets were cut into cubes and immersed in cold 2.1% sucrose overnight. Cubes were then frozen in liquid nitrogen on pins. Sections were cut from a block, next 20 mm were cut, then the block was resectioned. Ultrathin cryosections were cut on a UC7 cryo-ultramicrotome (Leica Biosystems) and contrasted with a mixture of 3% uranyl acetate and methyl cellulose. Separate areas of the three blocks, 12 in total, were sampled, and approximately 40 to 50 β-INSU expressing cells in total were examined. INSU IgG rabbit antibody was visualized in the L120C transmission electron microscope (FEI Hillsboro) with either 10 or 15 nm protein A-gold. Sections were incubated with INSU and LAMP1 mouse antibody for 30 min. LAMP1 labeling was performed with a rabbit anti-mouse bridging antibody and visualized with either 5 or 10 nm protein A-gold. Double labeling was performed in sequence. The protein A-gold secondary was used alone as a negative control (79). Structures were scored as positive if there were 5 separate gold particles on the inside or outside of the structure. No mature granules scored as positive. Occasionally, single gold particles were found on the mature granules and were identified as background. Random sampling of three 1 mm cubes per pellet was performed to examine as many islets as possible.

### Sample preparation and MS-based SRM

The proteotypic peptide selection was based on empirical procedures that balance ideal attributes of the assays with practical limitations - for instance, the tryptic peptides available from the INSU (23). The selected peptides were produced as synthetic stable isotope (heavy) labeled and unlabeled peptides (light) by Genemed Synthesis Inc. After reconstituting, the concentration of each synthetic peptide was determined by amino acid (AA) analysis (New England Peptide, Gardner).

Selection of optimal charge state, SRM parameters (*i.e.* DP, CE, and CXP), confirmation of coelution of endogenous and SIS peptides, and interference detection were conducted as detailed elsewhere (80). All samples from islets (selected ∼100 islets each from eight (21) T2DM donors and 15 control donors), plasma (5 ml), and CSF (250 ml) were analyzed on a 5500 triple quadrupole (QTrap) mass spectrometer (Sciex) using Analyst software version 1.7.2. Subsequent data processing was performed using MultiQuant software (Sciex, version 3.02 with Scheduled-MRM-Algorithm). Three to six interference-free SRM ion pairs constituted the final SRM assay for the respective proteotypic peptides, pep-U1, (detects all INSU isoforms) -U2 (detects INSU1, U2, and UC isoforms), -U4 (detects INSU1 and U2 isoforms), and -US (detects INSUB isoform), we did not select pep-U3 and -U5 because the redundancy since they are connected to pep-U2 and -U4 respectively (14). For further enhancement of SRM sensitivity, we scheduled the mass spectrometer to collect subsets of peaks based on the target analyte retention times (RT) on the column. Compared with classical SRM modes, the scheduled SRM provides amplified signal-to-noise due to higher dwell times, and greatly improved reproducibility and accuracy by detecting more data points across chromatographic peaks. We previously published details of SRM quantitative validation (14). SRM data points shown in the figures were biological replicates.

### Cycloheximide and bafilomycin treatments of human islets

*Postmortem* human islets were obtained from IIDP and Prodo Laboratory Inc. Donor information is provided in supplementary Table S3. The islets were delivered in PIM(S) islet specific medium (Prodo Lab). After arrival, human islets were centrifuged at 500 g for 5 min, and the pellet was resuspended in PIM(R) medium (Cat# PIM-R001GMP) supplemented with human AB serum (Cat# ABS001GMP). The islets were rested at 37 °C overnight and then washed three times with PIM(R) without human AB serum which would interfere with downstream SRM assay. Two independent batches of islets were treated with cycloheximide (that blocks protein elongation) or bafilomycin (a lysosome inhibitor that blocks V-ATPase) in serum-free PIM(R) medium. We used (1); 10 μM cycloheximide (Thermo Fisher Scientific, Cat# J66901.0: stock1 mM in ethanol) (2, 81) 0.1 μM bafilomycin A1 (Cat# J61835.MCR: stock 0.1 mM in DMSO) (82), and (3) ethanol (1%) and DMSO (1%) as control vehicles for cycloheximide and bafilomycin A1, respectively. We used LIVE/DEAD Cell Imaging Kit (Cat# R37601) to measure human islet viability at different time points. We observed that the islet cell death/live ratios were similar up to 2 h but by 4 h the ratio of death to life cells had increased by 40% (Fig. S5, *A* and *B*), so we did not include the 4 h point in further experiments. After centrifugation, the islets were harvested and washed with 3 ml of 1XPBS (without Ca^2+^ and Mg^2+^). The pellets were then homogenized in 100 μl of 0.2% RapiGest SF (SKU: 186002122, Waters, Milford, MA) containing 100 mM Tris-HCl, pH = 8.0, sonicated twice for 5 s with a 30-s interval on ice, and centrifuged at 14,000 rpm for 20 min at 4 °C. From the resulting supernatant, 5 μl was used to measure protein concentration with Qubit Protein Assay Kit (Cat# Q33211) for normalization of SRM assay, and the remaining volume was used for downstream SRM assay.

### Statistical data analysis

GraphPad Prism v9.0.1 software was used for statistical analysis and data are presented as means ± SEM for RT-ddPCR data and ±SD for SRM data. The normalized expression values of INSU positive ddPCR droplets, SRM quantitative data, and INS-VNTR association were analyzed using a two-tailed unpaired Student *t* test, one-way ANOVA, and Chi-square test, respectively. The mean ratio of INSU in plasma and CSF was estimated by error-propagation. Spearman correlation coefficient analysis was performed for INSU SRM linear regression for glycated hemoglobin, body mass index, and age of the BLSA participants’ plasma samples. Multiple variables of INSU peptides were analyzed using partial least squares-discriminant analysis and the accuracies were calculated by area under the receiver operating characteristic curve (ROC). *p* < 0.05 was considered significant.

## Data availability

The data resources (TaqMan probes, INSU antibody, and SRM-MS peptides) generated and/or analyzed during the current study are available from the corresponding authors upon reasonable request.

## Supporting information

This article contains supporting information.

## Conflict of interest

The authors declare that they have no conflicts of interest with the contents of this article.
