# Supplementary material for: Cell type-specific expression and subcellular localization of the human insulin upstream open reading frame (INSU) protein in pancreatic β-cells
Source: J Biol Chem. 2026 May 29;302(7):113209. doi: 10.1016/j.jbc.2026.113209 (PMC13314798; doi:10.1016/j.jbc.2026.113209)
Supplement: Supporting Figures and Tables [file mmc1.docx]

**Supporting Information**

**Cell type-specific expression and subcellular localization of the human insulin upstream open reading frame (INSU) protein in pancreatic β-cells**

Qing-Rong Liu^1^*, Min Zhu^1^, Lisa M. Hartnell^1^, Jane Tian^1^, Qin Yao^1^, Xiaoming Zhong^2^, Chee W. Chia^1^, Paritosh Ghosh^1^, Máire E. Doyle^1^, Jennifer F. O’Connell^1^, and Josephine M. Egan^1^*

1 NIA/IRP/NIH, Baltimore, 251 Bayview Blvd, Baltimore, Maryland 21224. USA.

2 Center of Excellence for Leukemia Studies, St. Jude Children's Research Hospital, Danny Thomas Place, Memphis, TN 38105, USA

* Correspondence: Qing-Rong Liu (qliu@mail.nih.gov) and Josephine M Egan (eganj@grc.nia.nih.gov), LCI/NIA/NIH, 251 Bayview Blvd, Baltimore, Maryland 21224, USA.

Key words: Insulin, Islets, Proteomics, Diabetes, Crinophagy, Evolution

**Running title:** Human-chimp-specific insulin uORF in pancreatic β-cells


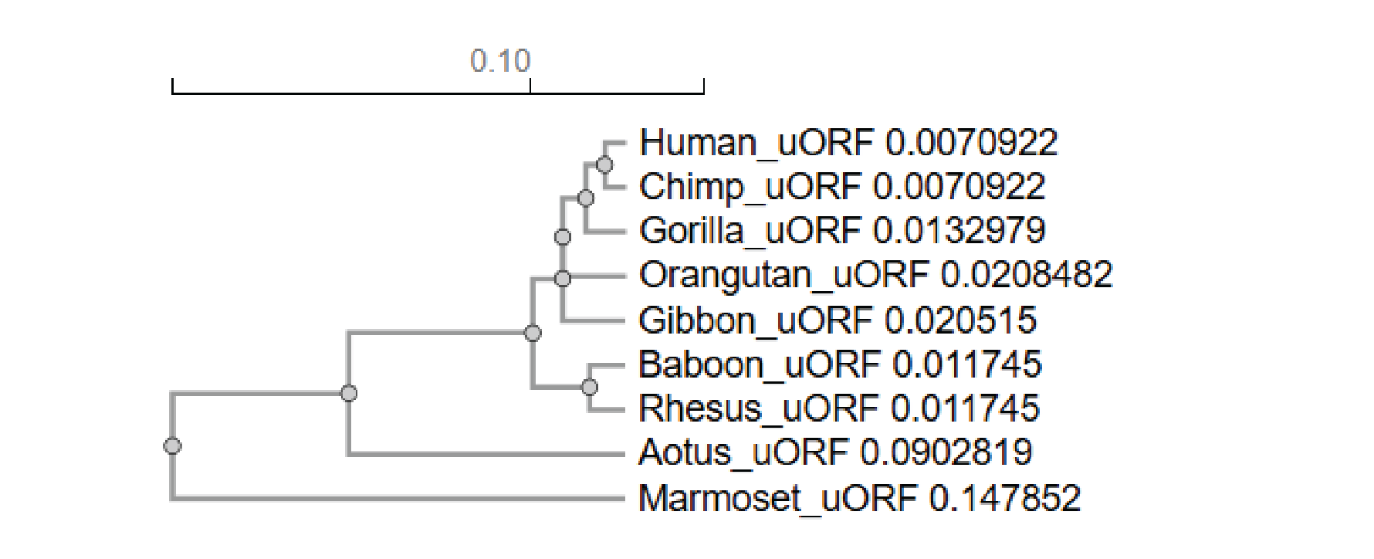


**Supplemental Figure S1. Phylogenetic tree of INSU1 uORF**. Branches represent lineages and nodes speciation events. Numbers represent rates of nucleotide substitutions of the primate uORFs.


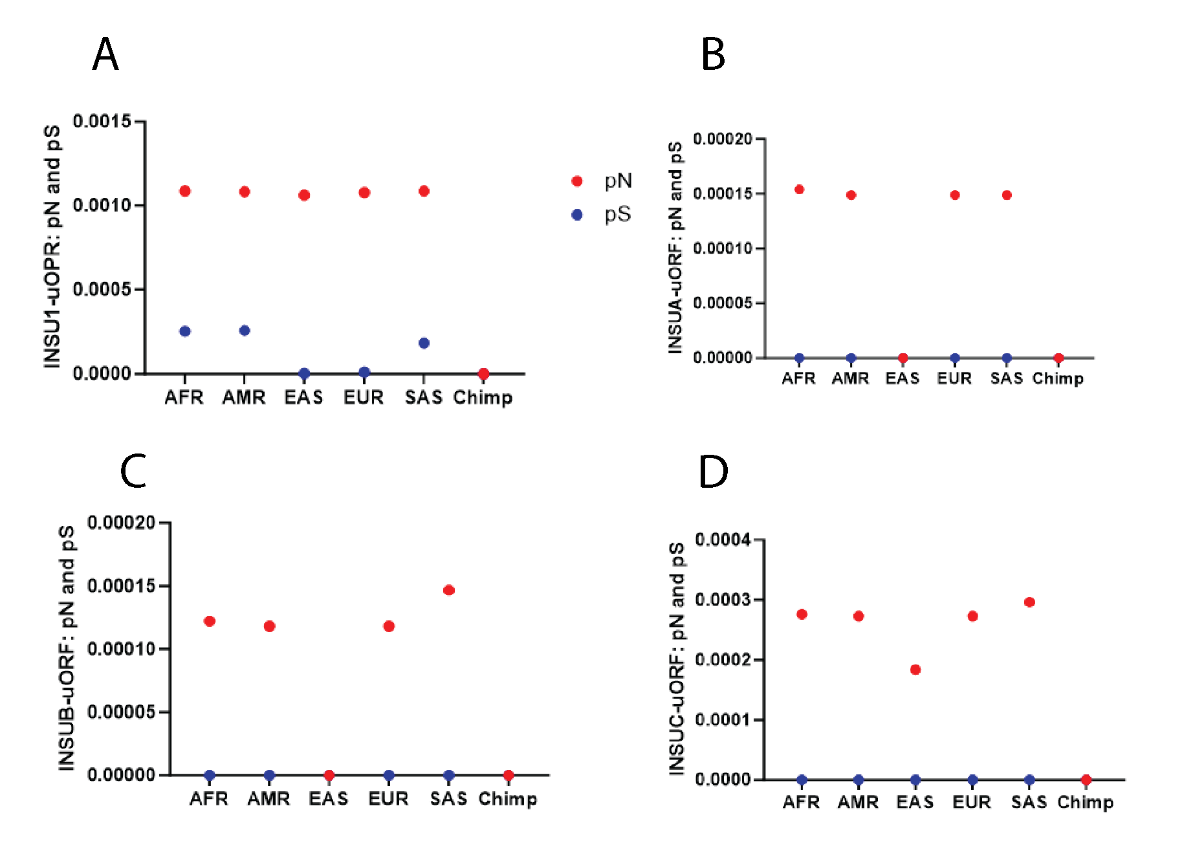


**Supplemental Figure S2. Scatter plots of pN and pS for INSU isoform uORFs in different human populations and chimp.** AFR represents African; AMR Admixed American; EAS East Asian; EUR European; SAS South Asian. *A*, INSU1, *B*, INSUA; *C*, INSUB; and *D*, INSUC.


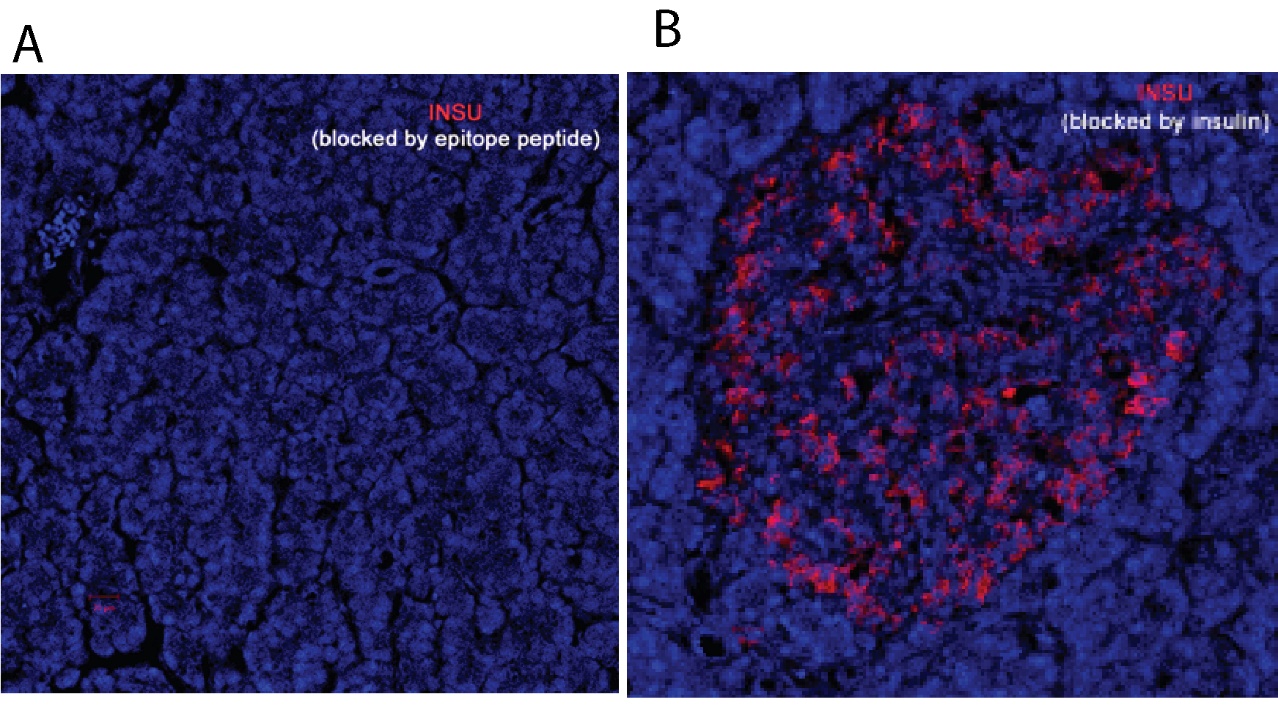


**Supplemental Figure S3.** **Immunofluorescence (IF) labeling of INSU rabbit polyclonal antibody in pancreatic islets.** *A*, INSU antibody was blocked by the antigenic epitope peptide. *B*, INSU antibody was not blocked by the mature insulin.


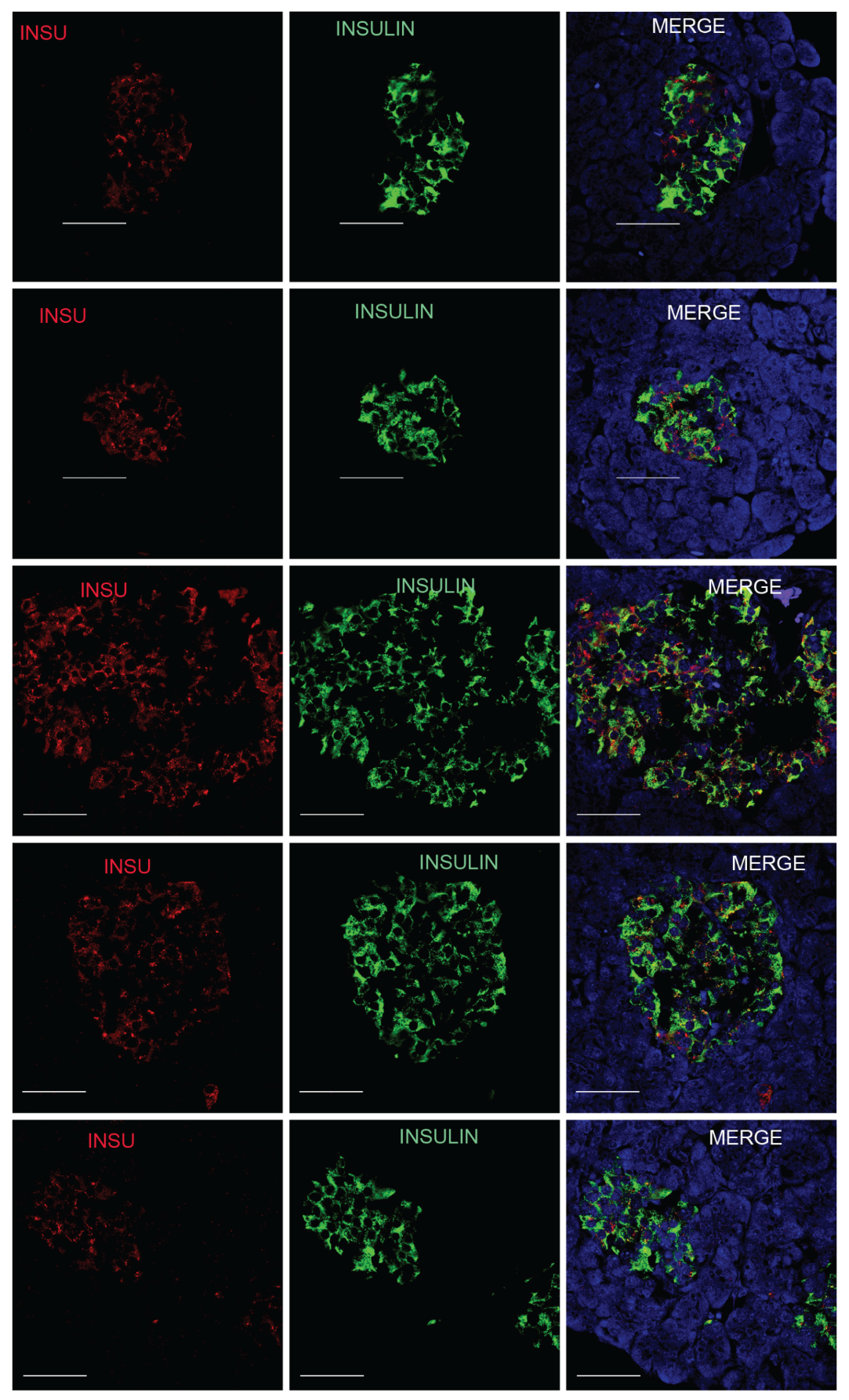


**Supplemental Figure S4.** Representative islet IF images used for HALO AreaQuant analysis.

**uORF translation initiation INSU Epitope**

**INSU1 MGSETIKPAGAQQPSALQDRLHQKRPSSRSVPRAFASGGLRIPGWLDPRPQLCSREDVAG 60**

**Ins1 LPTHPTWRP-WAKQQSPGGREEVLWTIKLVGIQ-PPALSDQL-SETISKQVCTLLFGPGS 57**

**Ins2 LLTYPTWSP-WVKQQSPGGREEVLWSIKVVGTQ-PPALSDPLQSKTISKQEGTLLSGPGS 58**

**: : : .: .* . . : : . : . : . ..**

**pORF translation initiation**

**INSU1 LVKHVGVSPGAPRQGTWPSACLSPACLPDHCPSAMALWMRLLPLLALLALWGPDPAAAFV 120**

**Ins1 PAKTPATLGRMWAPLLHGSFASLNPAYLSGHCFNMALLVHFLPLLALLALWEPKPTQAFV 117**

**Ins2 PAKTSGTGR--IPPLLRETFAILNPAYLPGYCFNMALWMRFLPLLALLFLWESHPTQAFV 116**

**.* .. : . . *** :::******* ** .*: *****

**INSU1 NQHLCGSHLVEALYLVCGERGFFYTPKTRREAEDLQ 156**

**Ins1 KQHLCGPHLVEALYLVCGERGFFYTPKSRREVEDPQ 153**

**Ins2 KQHLCGSHLVEALYLVCGERGFFYTPMSRREVEDPQ 152**

**:***** ******************* :***.** ***

**Supplemental Figure S5. Amino acid sequence alignment of human INSU1 and corresponding regions of mouse Ins1 and Ins2.** The upstream open reading frame (uORF) translation initiation methionine is marked by red M, and the primary open (pORF) translation initiation methionine is marked by blue M, the translation stops of uORF are marked by red dash lines in mouse Ins1 and Ins2, identical amino acids by asterisks, conservative substitutions colons and semiconservative by dots.

**Supplemental Figure S6.** LIVE/DEAD™ Cell Imaging of human islet viability. (A) Viability assay of control and cycloheximide treatment at 0.5, 1.0 and 2.0 hr. (B) Viability assay of control and bafilomycin treatment at 0.5, 1, 2, and 4 hr. Red represents dead cells and green live cells.

**Supplemental Table S1.** The pN and pS of human and chimpanzee polymorphism in *INS* gene loci was estimated by SNPGenie for INSU1, INS, uORF of INSU1, INSUA, INSUB, and INSUC coding sequences in different human populations and chimps. The numbers of genomes and SNPs are shown on the header.

**Supplemental Table S2.** Clinical data of BLSA plasma samples

**Supplemental Table S3.** The human islet samples for cycloheximide and bafilomycin treatments.
